# Monitoring-Based Model for Personalizing the Clinical Process of Crohn’s Disease

**DOI:** 10.3390/s17071570

**Published:** 2017-07-05

**Authors:** Alberto de Ramón-Fernández, Daniel Ruiz-Fernández, Diego Marcos-Jorquera, Virgilio Gilart-Iglesias, Víctor Vives-Boix

**Affiliations:** Department of Computer Technology, University of Alicante, Alicante 03690, Spain; aderamon@dtic.ua.es (A.d.R.-F.); dmarcos@dtic.ua.es (D.M.-J.); vgilart@dtic.ua.es (V.G.-I.); vvives@dtic.ua.es (V.V.-B.)

**Keywords:** personalized health, health monitoring, artificial intelligence, Crohn’s disease

## Abstract

Crohn’s disease is a chronic pathology belonging to the group of inflammatory bowel diseases. Patients suffering from Crohn’s disease must be supervised by a medical specialist for the rest of their lives; furthermore, each patient has its own characteristics and is affected by the disease in a different way, so health recommendations and treatments cannot be generalized and should be individualized for a specific patient. To achieve this personalization in a cost-effective way using technology, we propose a model based on different information flows: control, personalization, and monitoring. As a result of the model and to perform a functional validation, an architecture based on services and a prototype of the system has been defined. In this prototype, a set of different devices and technologies to monitor variables from patients and their environment has been integrated. Artificial intelligence algorithms are also included to reduce the workload related to the review and analysis of the information gathered. Due to the continuous and automated monitoring of the Crohn’s patient, this proposal can help in the personalization of the Crohn’s disease clinical process.

## 1. Introduction

According to the World Health Organization (WHO), chronic diseases are by far the leading cause of mortality in the world, representing 60% of all deaths. They are diseases of long duration and generally slow progression. In 2008, 36 million people died from chronic diseases, 29% were under 60 years old and half were women [1]. The lives of patients with chronic diseases is usually affected because of treatments or changes they must instill in their daily routines to ensure the preservation of quality of life. Another important issue related to this kind of disease is the cost associated with treatments; they are usually expensive and patients need them for the rest of their lives.

Crohn’s disease produces inflammation of the gastrointestinal tract and it can affect any area from the mouth to the rectum. This pathology can affect people of any age, but is more common in people between 15 and 35 years years of age. At present, Crohn’s disease has an unknown origin, although it is known that family history and some toxic habits such as tobacco or alcohol and poor diet are risk factors [2,3]. A major disadvantage of this disease is the number of symptoms that it shares with other types of pathologies, which makes its diagnosis difficult (the most common symptoms are abdominal pain, vomiting, diarrhea, weight loss, infections, bleeding, etc.). A Crohn’s patient should be monitored by different medical specialists, depending on what kind of complications arise. In the most acute cases, a surgical intervention is necessary to remove the part of the damaged intestine and prevent a major infection. The treatment for Crohn’s patients is composed of pharmacological treatment and measures aimed at leading a healthy lifestyle. Factors that positively affect disease control include maintaining a balanced diet with good eating habits, avoiding alcohol and tobacco consumption, performing physical activity at least four times a week, avoiding stressful situations and respecting the quality of sleep.

We focus our research in achieving a monitoring-based model for personalizing the clinical process of Crohn’s disease centered on two main goals. First, our proposal should help medical staff to know, in a more detailed and continuous way, data from clinical variables and daily routines that can be useful to personalize the treatment of a Crohn’s disease patient. To do that, we base our model on a distributed system which includes data monitoring (weight, physical activity, blood pressure, mood, fecal calprotectin, and stools status and frequency) from the patient and send it to the physician. In addition, and to improve the personalization, we also include the monitoring of ambient variables, such as the ultraviolet (UV) index. Thanks to these data, the physician can adjust the treatments to the specific characteristics of a patient. The second goal is to reduce the associated costs to the monitoring and treatment of a patient with Crohn’s disease, preserving the quality of the health care. In order to reach this objective, it will be necessary to incorporate artificial intelligence algorithms that can automate the process of taking decisions [4]. In this way, clinical staff can be released from mechanical activities of data analysis. This task is aimed to avoid, for example, exacerbations that can imply a hospital admission with the corresponding extra cost and a loss of quality of life for the patient.

The next section will show the state-of-the-art technology used to personalize health care and the technical developments to help patients with Crohn’s disease. In Section 3, we will present the clinical process of the Crohn’s disease together with some deficiencies in the process that our proposal could address. Next, we will explain the proposed model as well as a redesign of the clinical process. Then, in order to make the model viable, an architecture will be presented, focusing on the monitoring the variables and the artificial intelligence module to automate the data analysis and decision-making process. In Section 6 we present a prototype as an example of implementation and how the proposed system would work in a test scenario. Finally, we share the conclusions for our proposal.

## 2. Background

In recent years, the fast evolution of information and communication technologies (ICTs) and their impact on society has also been reflected in the field of health (eHealth). Improving care for chronic patients represents one of the main challenges for health professionals and focuses much of their efforts. Real-time patient monitoring and personalized medicine are seen as the future in this field. In order to provide a continuous service, which improves the quality of life of the patient and reduces the cost borne by public health systems, a wide variety of mobile applications, web services and monitoring devices focused on meeting this demand have been arisen. Next, we describe some of them to show how these have helped in chronic patient care. This is the case of studies performed by Kollmann and Pinsker [5,6] who developed different applications to treat diabetes. In them, the patient, through the mobile app, introduces diabetes-related data and this information is sent directly to a data center, where it is stored and processed to generate graphs, trends, reminders and other messages. Other chronic diseases affecting a greater number of patients are chronic obstructive pulmonary disease (COPD) and hypertension. The mobile app designed by Vorrink et al. [7] aims to encourage the practice of physical exercise for COPD patients. In this case, the accelerometer of the mobile is used to calculate the physical activity of the patient and to compare it with predefined goals.

On the other hand, Agarwal et al. [8] defined a monitoring system for the hypertensive patient. Through a mobile device, the patient enters data related to the blood pressure and sends this information to an online server. Physicians can access to the clinical information through a web portal and send their feedback to the patients. Moreover, automatic alerts are sent if the blood pressure limit is exceeded. It was also proposed to use this app for monitoring diabetes, including the measuring of glucose with a glucometer that could be coupled to the phone. Cardiac diseases also affect a large number of patients and different studies have focused on their follow-up. In this regard, a home monitoring system was developed for the follow-up of patients affected by various types of heart diseases [9]. Patients send their weight and blood pressure data through a mobile device to the telemonitoring server. Physicians can consult via web patient information such as weight, blood pressure, date of last measurement, vital signs, medication and events, and they are notified if any patient exceeded predefined limits. Thus, the physician could set automatic reminders, alerts, medication, etc.

Regarding Crohn’s disease, different studies support both the advantages of personalizing the treatment for patients with inflammatory bowel disease [10], mainly at a pharmacological level with the use of pharmacogenomics approaches, and the feasibility of telemedicine to improve treatment adherence and patient self-management [11]. This is achieved through systems that allow for communication between physicians and patients, symptoms control and improving the information provided to the patients.

However, there are not many studies focused on improving the clinical process trough eHealth. To this end, Pedersen et al. [12] assessed the effectiveness of a web service for improving patients’ adherence to treatment. The patients recorded their disease activity and fecal calprotectin (a non-invasive marker to discard false positive results) weekly. With these parameters, a burden of inflammation and an objective marker of inflammation are calculated. This marker classifies the patients according to the degree of the disease in three different bands differentiated by colors (green, yellow and red). If the patient is in the yellow or red band, the patients are referred to an infliximab (IFX) infusion. As a conclusion, the web service appeared to be a practical and safe concept for the scheduling of IFX treatment. On the other hand, Vinding et al. [13] have developed *CalproSmart* test method, a new tool to measure the fecal calprotectin. It consists of a camera smartphone app and an extraction kit. The patient takes a sample of their stools and mixes them with a solution that will be deposited in the sample window. The sample window shows a test line and control line. The patient takes a picture of the sample window using the app on their smartphone, and the ratio between the two lines’ staining intensity is calculated and processed by a data center. Finally, according to this ratio, the patient receives either a green, yellow, or red dot on their smartphone, which represents the disease activity (none, moderate and high activity respectively).

More recently, in the study of Con et al. [14] a global view on the use of eHealth with Crohn’s patients is provided. They have explored the content and tools of existing apps to identify functionalities that may facilitate patient self-management. Most of these apps present several weaknesses such as lack of standardized clinical guidelines, lack of involvement of medical staff and none of them offers support to facilitate self-initiation for medical therapy. This represents one of the most important shortcomings of the current systems: the scarce support physicians have to diagnose and define a treatment as personalized as possible to the characteristics of the patient. In this regard, clinical decision support systems (CDSS) represent an important progress in eHealth. These systems are capable of evaluating patient characteristics and provide accurate recommendations for the treatment, monitoring and diagnosis of the disease. Thus, they become an aid tool to facilitate the diagnosis and treatment to the clinical staff. To better analyze their impact, Roshanov et al. [15] performed an evaluation of the decision systems in the most common chronic diseases (diabetes, hypertension, COPD and cardiac care). The study showed that most of these systems improve patient care processes and in some of them the improvement of the patient’s health is achieved. However, the effectiveness of these systems should still be tested more consistently with larger study populations. Most of these decision support systems, such as the one developed for the COPD process [16] are useful to remind the patient to take the prescribed medication, dose changes, alternative treatments or other specifications. The operation of these CDSS is usually based on algorithms or conditional (*IF-THEN*) rules based on patient-specific data and on the clinical guidelines developed. Furthermore, standardization of clinical guidelines has been shown to be effective in improving the concordance of clinical decisions in multidisciplinary teams as in [17] where, based on rehabilitation programs previously defined, the CDSS allows for evaluation of the most suitable program, according to the characteristics of the patient and the type of heart disease.

In [18], the CDSS searches an electronic data record of hypertensive patient vital signs and lists of problems, medications and allergies to determine if the patient was receiving the correct medication according to the status of the disease and based on the clinical guide. This system automatically generates recommendations based on measurements of blood pressure, age, sex, race for both the physician and the nursing staff for each problem using the documentation of the disease. These systems also seek to improve adherence to treatment on the part of the patient, as in [19] where a CDSS guides the patients in their treatments through monthly alerts and reminders.

However, although decision support systems are being integrated into the common chronic processes, no Crohn’s disease studies involving decision support systems have been found. The lack of knowledge about this disease, which affects a smaller number of patients than other common chronic diseases, prevents patient self-management, increasing the number of visits to health centers and the cost borne by public health systems. Furthermore, the large number of symptoms it shares with other diseases makes difficult its diagnosis by the physician. Therefore, a clinical decision support system that assists the physician for the diagnosis and treatment of the patient would improve the effectiveness of the care process, reducing cost and improving the patient’s quality of life.

## 3. Crohn’s Disease Clinical Process

**Figure 1:**
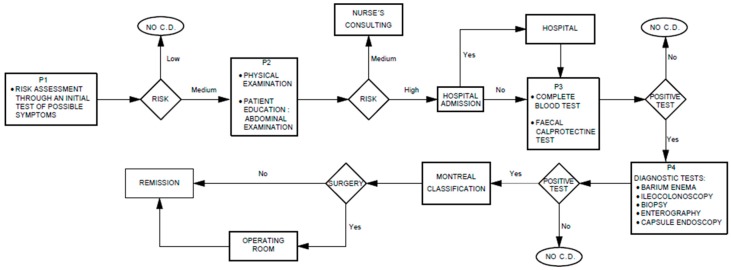
Current diagnosis and treatment Crohn´s disease process.

In this section, the clinical process of Crohn’s disease is defined from its diagnosis until its treatment. The main goal is to understand how the process is currently developed (AS_IS view) and how many factors are involved. The analysis carried out in this work is supported by international clinical guides (Canada, United Kingdom) [20,21]. The procedure starts with the first clinical evaluation performed by medical and nursing specialists (see P1 in Figure 1). In case of medium-high risk, a doctor will perform a physical exam supported by an abdominal X-ray, blood pressure values, heart rate and temperature (see P2 in Figure 1). The third stage of the diagnosis implies the valuation of the specialist doctor in digestive system. The doctor will carry out a complete analysis as well as the fecal calprotectin test. This test allows the specialist to discard false positive results (see P3 in Figure 1). Finally, the patient is subject to several tests carried out by different health specialists. If any of them are positive, a Crohn’s disease diagnosis is confirmed (see P4 in Figure 1).

Once the patient is diagnosed with Crohn’s disease, the grade of the disease is defined. In that moment, it will be decided if the patient needs a surgical intervention or they are derived to the induction stage, where they are monitored. In the case whereby the patient needs a surgical intervention, the possibility of deriving the digestive tract to a stoma is studied. Therefore, whether the surgical intervention is performed or not, the patient will be continuously evaluated in the follow-up stage. In this phase, the tests related to the diagnosis are performed again with the purpose of looking for possible complications and reclassifying the disease degree of the patient.

### Weaknesses in the Current Crohn’s Disease Clinical Process

At present, the management of the Crohn’s disease clinical process presents important deficiencies both in the diagnosis and in the treatment phase. One of the main problems of the treatment is the lack of standard clinical guidelines and the time it takes to be diagnosed. Usually, the fecal calprotectin test is carried out only in the final step of the diagnostic phase [22]. The lack of psychological support during the process is among the most significant deficiencies [23]. From the point of view of the patient, factors such as the continuous visits to primary care centers, absenteeism and the large number of tests to perform have affect the quality of life [24]. Another important aspect negatively affecting the quality of life is the lack of clear and updated information throughout the clinical process [25]. The annual cost of caring patients with Crohn’s is one of the aspects having more relevance throughout the process. The hospitalization rate is increasing and it represents a significantly higher cost than keeping the patient in remission. In [26] it was estimated that the costs related to surgery accounted for about 40% of the total costs of the process. We will redesign the clinical process, implementing the necessary improvements, and minimizing most of the deficiencies identified, using ICTs as a key element to achieve a high personalization degree for monitoring and treatment of the disease.

## 4. Proposed Model

**Figure 2:**
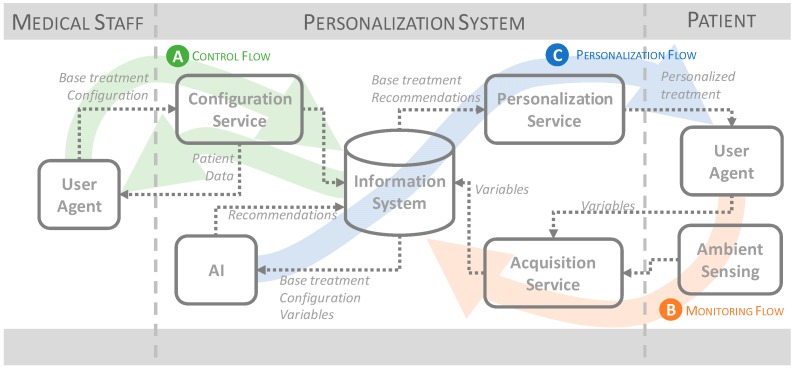
Proposed model.

The main objective of our proposal is providing patients a better health service thanks to the personalization of the health care. In general terms, this means an unacceptable increase in workload for the medical staff because they should treat each patient individually. In our proposal, we use information technologies to allow for individual monitoring and treatment using personal and ambient sensors, an automatic analysis of information and a set of recommendations for the patient, supervised by the doctor. A general view of the model can be observed in Figure 2.

The general objective for the model has been mentioned: it is focused on the personalization of the health care of a Crohn’s patient; together to this objective we want to reach a set of secondary objectives, necessary for the correct working of the system.

- Automation
- Twenty-four hour monitoring
- More certainty in diagnosis and treatment
- Low cost

These secondary objectives are interrelated. For example, for 24-h monitoring, it is necessary to have an automated system; any other way, it would be unacceptable in monetary terms. The health of the patient must be monitored constantly; it is only in this way it is possible to personalize their health care and doctors will have continuously a great detail about the health of the patient. Thanks to that, health information about a patient, changes in diagnosis and treatments will be more precise and doctors will give their opinions with more certainty; furthermore, the recommendations or opinions from the doctors will not be general but specific for a particular patient. Finally, if we want a viable system from the proposed model, it is necessary that it be a low-cost system or, at least, that savings due to benefits were higher than expenses for implementation and maintenance. In that case, it is important to bear in mind that we face a chronic disease, and any savings will be for the rest of the patient’s life. Furthermore, as we have mentioned above, automation in the system will save money because, for example, regular appointments for monitoring can be avoided (patients could do the calprotectin test and the results are sent to the doctor).

Although the design of the proposal has taken into account the features of the Crohn’s clinical process, the resulting model is sufficiently general and flexible enough to be applied to other diseases.

In our proposal, the personalization process is done through the concept of recommendations. A recommendation is an action that the proposed model suggests to the patient so that it is incorporated into the process of treatment of the disease. These recommendations vary over time, depending on the treatment of each patient, and the evolution observed in the monitoring, obtained from the capture of a set of variables related to both the patient and the environment.

To carry out the personalization process, the model is based on three flows of information working in the global system in a decoupled and independent way, where the information system acts as a common link between the three flows.

### 4.1. Control Flow

The control flow (A in Figure 2) is supported by actions taken by medical staff. Fundamentally, it consists in the establishment of a base treatment for each patient and some configuration parameters that will guide the process of personalization. Through this flow, the medical staff is also able to perform a complete process monitoring, obtaining all information pertaining to each patient, including the variables monitored and the recommendations resulting from the personalization process.

The elements involved in this flow are: user agents that allow professionals to access information and that can be implemented as web, mobile or desktop applications; a configuration and monitoring service, which acts as the only point of entry for user agents to the system and that offers the functionality and information necessary to carry out this flow; and the information system where the configuration established by the professionals is stored and from where the patient data are obtained.

### 4.2. Monitoring Flow

The monitoring flow (B in Figure 2) allows the system to periodically obtain information, both from the patients and from their environment, relevant to the treatment of their disease. The monitoring of this information is fundamental for the model, since it supposes the knowledge base necessary for the personalization process of the treatment. To do this, the patient will have his own user agent to introduce, either automatically or manually, physiological variables such as weight, lifestyle variables (such as physical activity or mood), and symptomatic medical variables such as the presence of fever or headache. In addition, in the patient’s environment, there will also be an environmental sensing system, which will collect environmental variables associated with the disease, such as the UV radiation index.

The elements involved in this flow are: user agents and the environmental sensing system, as elements that capture the monitored variables; an acquisition service, capable of receiving the monitored information, processing it and normalizing it; and the information system, where these variables are stored for later consultation and analysis.

### 4.3. Personalization Flow

The last flow, the personalization flow (C in Figure 2), aims to create personalized treatment for each patient. For this, a module based on artificial intelligence obtains all the information concerning the patient as well as the basic treatment and the configuration made by the medical staff, and based on these data generates and stores the recommendations in the information system. These recommendations will change as the input data changes. Subsequently, a personalization service will collect these recommendations and, together with the base treatment, will offer the patient this information in an appropriate way by means the user agent.

### 4.4. Redesign Process

**Figure 3:**
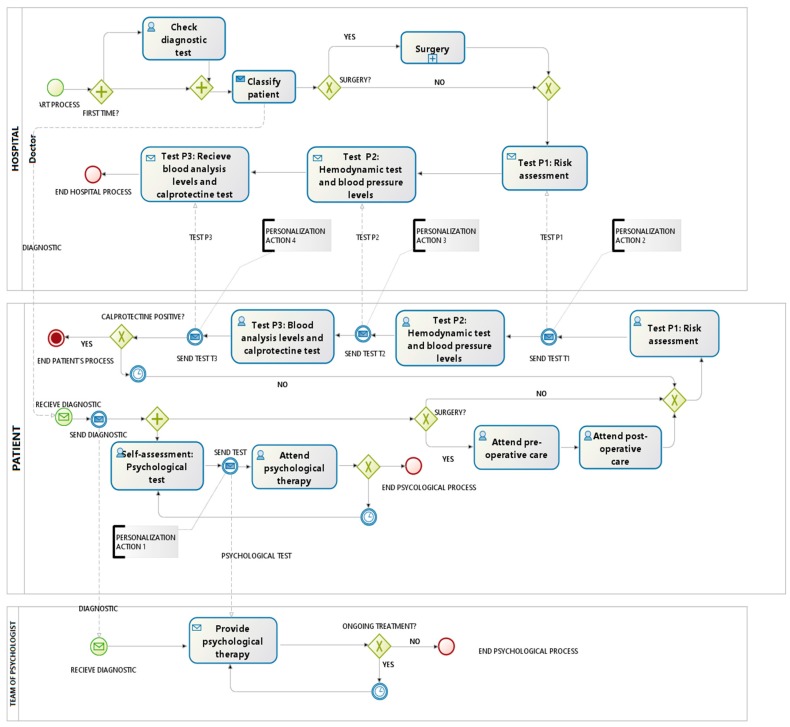
Proposed redesign process.

Once we have detected the weaknesses and with the aim of personalizing the monitoring and treatment, we focus on the redesign of clinical Crohn’s disease process using Business Process Management Notation (BPMN). Using the model proposed (see Figure 2), we redesign (see Figure 3) the current process, including actions that could help to improve the personalization of the diagnosis and treatment; for example, we include a phase of psychological support to the patient that can help to do recommendations for a specific patient. Through specific user applications integrated with the system by means of web services, the patient can make self-assessment tests which will be integrated into the system in order to be analyzed by the team of psychologists (personalization action 1).

Through web services, a patient can also send information related to various risk factors, blood tests values and other useful information referred to physical examination (personalization action 2). In addition, data concerning about weight, blood pressure and physical activity are incorporated automatically to the system thanks to the connection with smart devices (personalization action 3). To improve the control over a patient and to rule out misdiagnosis, fecal calprotectin can be measured and the information integrated with the rest of information; furthermore, together with these measures, data about stools (such as frequency or consistency) is also useful for the monitoring of a patient (personalization action 4). Thus, the medical team has an updated medical history, improving the control over a patient; this control turns into an improvement of quality of life for the patient and a reduction of costs for the health system.

## 5. Proposed Architecture

**Figure 4:**
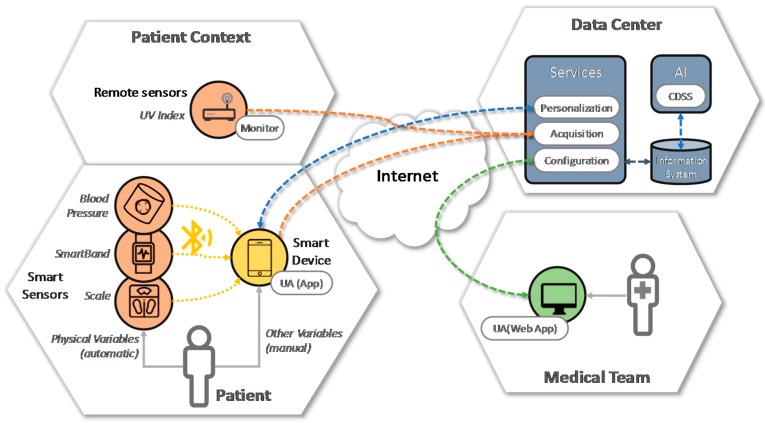
System architecture for Crohn´s disease management.

Once we have modeled our proposal to achieve a personalized monitoring and treatment of the disease, we have to define how this model can be implemented. In order to do that, we present an architecture (see Figure 4) with three main areas: patient, medical team and data center. The objective of the architecture is to instantiate the model, specifying the elements that make it up and defining the functionalities of each element, how these elements are distributed in a scenario and the relationships between them. These areas are connected through the internet, so that, all the information related to the patient flows continuously between the distinct parts of the architecture, integrating the different flows or information types explained in the model proposed. It is important to highlight that we have designed the architecture bearing in mind new discoveries related with Crohn’s disease; for example, we have included a module for remote sensing in which ambient sensors or sensors out of the personal area are included. Next, the different areas and modules of the architecture are explained.

The patient’s area is focused on data acquisition in two ways, automatically and manually. There are clinical data that can be obtained directly from sensors such as weight or blood pressure; we say that this data is obtained in an automatic way thanks to the connection, usually using Bluetooth, of different devices (a scale, blood pressure monitor, smart band, etc. to a smart device (mobile phone, tablet, etc.) which are used as a gateway to send the information to a data center. These devices, used as gateways, are also the interfaces between patients and the system. Data from devices which are not connected directly to the system (for example, a scale without connection) is introduced manually through a user agent (such as an app) installed in a smart device (such as a smartphone). Data that are sent automatically can be also introduced manually through the user agent.

In the patient context we differentiate two parts according to the scope of the sensors (see Figure 4); we have sensors that belong to the personal area and they obtain measures in which a patient or an action of a patient is needed (physiological measures such as weight or patient’s activity measures steps in a day); also in the patient context we have sensors with data that can help the patients but data are not directly related with the patients: for example, information about UV radiation can be very useful for specific Crohn’s patients.

The area that belongs to the medical team includes the necessary user agents to interact with the system; this interaction can be done through a web application and it consists on the access to the data from the patient, the recommendations of the system and the configuration and changes of treatment or monitoring for a specific patient (for example, changes in the regularity of weight measures).

The third part is the core of the system composed by the information system, a set of services to give accessibility to sensors and user agents and a module based on artificial intelligence to support decisions and help doctors in the recommendations. All these different elements improve the control of the disease thanks to a closer monitoring and personalized recommendations from the doctors.

For the communication between all the distributed elements of the system, a service-oriented architecture (SOA) based on RESTful (REpresentational State Transfer) style web services is proposed. This service-based approach allows the integration of all elements with a weak coupling between them and brings fundamental features to the proposal such as reusability, autonomy, interoperability, composition and ubiquity.

The artificial intelligence (AI) module in the core system is based on a knowledge-based decision support system and it consists of three types of submodules: a knowledge base, an inference engine and a communication mechanism [27]. The knowledge base contains all the information (also called facts) that encode the expertise of the system about the application domain. This expertise is encoded in the form of rules and associations of compiled data, given beforehand by human experts, which take the form of IF-THEN rules. The inference engine is an automated reasoning system that evaluates the current state of the knowledge base, combines the rules with the current user’s data and then asserts new facts and knowledge to the knowledge base. The communication mechanism allows the system to collect information from the users, then shows them the results by evaluating their data in the form of personalized recommendations. The aim of the AI module is not to simulate an expert’s decision-making, but to assist doctors to personalize the decisions for a specific patient. The AI module, in the core system, is based on a knowledge-based decision support system.

## 6. Prototype

Following the guidelines explained in the architecture, we have implemented a prototype to monitor a patient with Crohn’s disease. We have selected different types of sensors to evaluate both ways of acquisition: automatically and manually. For the automatic acquisition, we use a scale connected with Bluetooth to a smartphone and a smart band to obtain the number of steps in a day as a measure of activity; for the manual acquisition, we introduce the characteristics of the feces and the calprotectin level with a calprotectin monitor; we have included also a UV sensor as a remote sensor.

This prototype has been implemented with the idea of working in a typical scenario of a patient with Crohn’s disease who must collect information about themselves, such as weight changes, daily activity, times and characteristics of stools; this patient should take care with the UV radiation (to avoid skin problems). Their doctor provides regular recommendations according to the information received.

**Figure 5:**
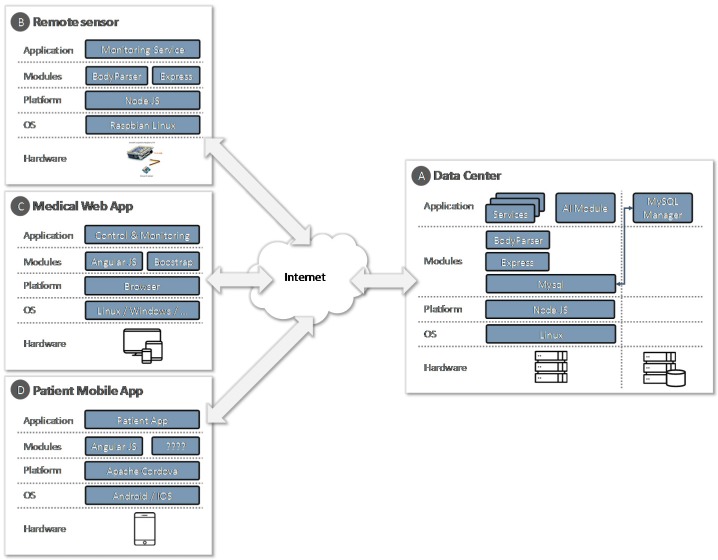
Technological architecture of system components.

For the design of the prototype, the service-oriented architecture pattern has been followed in a RESTful style. In Figure 5, a technological architecture for the prototype, with all its elements, hardware and software components, is shown.

Next, we will explain how we have implemented the different parts of the prototype.

### 6.1. Service and Storage

In the data center (Figure 5, block A), for the storage of the information, a *MySQL* database has been incorporated. For the development of RESTful style services, the Express framework on NodeJS has been used. In the implementation, the modules *MySQL* v2.13.0 and *body-parser* v1.17.1 have been incorporated. These services are consumed by the mobile application, the remote sensor and the web application used by the health center staff.

### 6.2. Data Acquisition from the Patient

**Figure 6:**
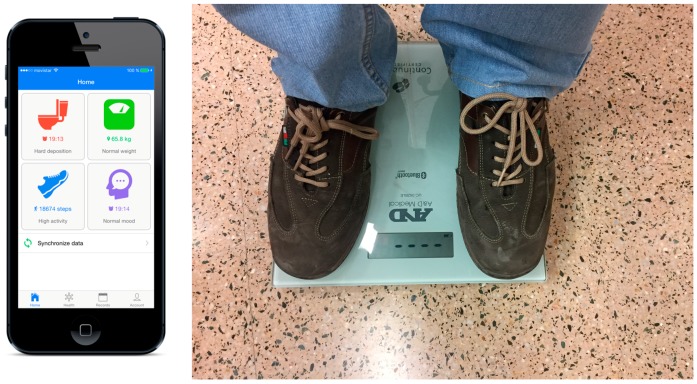
Technological architecture of system components.

The mobile application (Figure 5, block D), used as a data-acquisition platform, has been developed using *Apache Cordova* (Figure 6), a development framework that allows us to build applications for mobile devices by using web technologies (CSS3, HTML5 and JavaScript) instead of relying on platform-specific APIs like those in *Android*, *iOS* and *Windows Phone*. The mobile application has been developed following the basic principles of usability and support in clinical decision support systems [28].

Data can be acquired automatically and manually. Patient’s weight and blood pressure can be obtained via Bluetooth LE (Low Energy), also called Bluetooth Smart, from a digital scale and a digital blood pressure monitor of the A&D Company (Tokyo, Japan, *UC-352BLE* and *UC-651BLE* models respectively). In addition, the activity performed for each patient is collected from a *Fitbit Flex* smart band via their proprietary web API. Moreover, patients can also manually register their blood pressure and their weight (for cases where digital devices are not available), their fecal calprotectin results obtained with the calprotectin monitor, their daily mood and the status and frequency of their stools.

### 6.3. Data Acquisition from UV Remote Sensor

**Figure 7:**
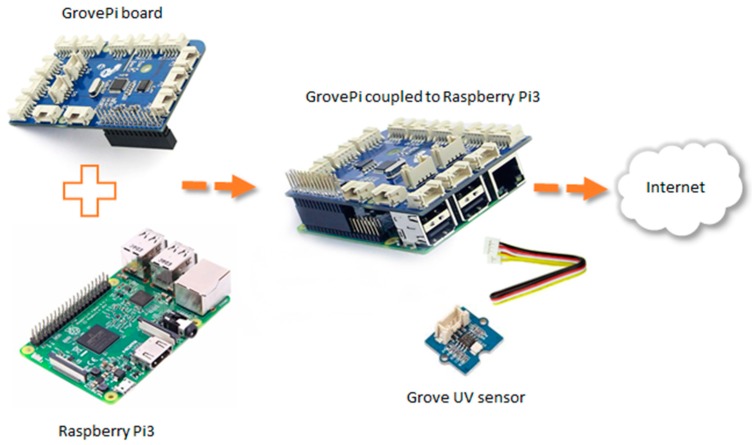
Hardware prototype for UV remote sensor.

To capture the intensity of ultraviolet radiation (UV) and to warn of the days where sun exposure represents a greater risk for the patient, a prototype for the measurement of ultraviolet radiation has been designed (Figure 5, part B). As it can be observed in Figure 7, the hardware architecture of our system is composed of three elements: UV sensor, computational platform and an add-on board. The *Grove UV sensor* is used to detect the intensity of incidents of UV radiation in an outside environment. It outputs electrical signals which vary with the change in UV intensity. To get information from the UV sensor, a single-board computer (SBC) called *Raspberry Pi 3* has been used as computational platform. *Raspberry Pi 3* includes integrated wireless LAN and Bluetooth, and allows the sending of data without the need to connect any other peripherals. The *GrovePi* board is an add-on board that is coupled to the *Raspberry Pi 3* and enables it to interface with Grove sensors avoiding soldering the sensor directly to the *Raspberry Pi 3.* The power of the system is done either through the electricity grid or a power bank due to its low consumption. Through a web service developed in *Node.js*, all the information collected by the sensor is sent to the data center for storage and management. For web services implementation, we also use the Express framework.

**Figure 8:**
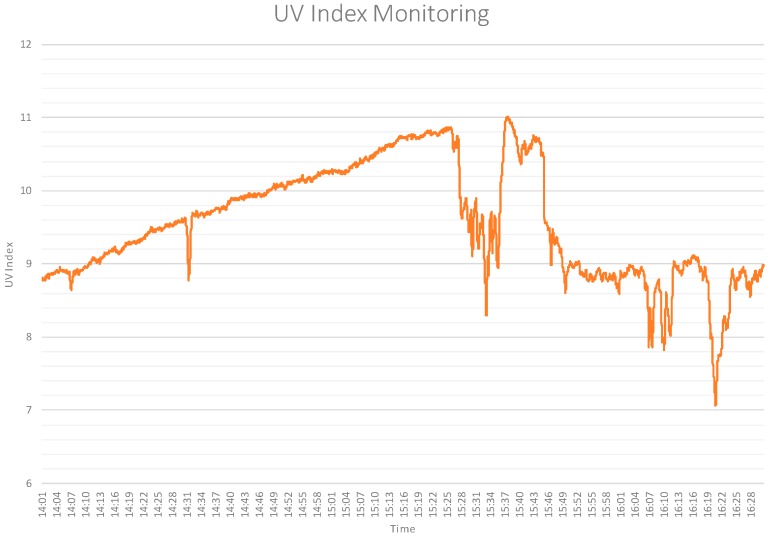
Evolution of the ultraviolet (UV) index.

To validate the adequacy of the proposal, several experiments have been carried out. In the first one, with the objective of performing a functional validation of the sensor, the prototype was placed outside a building, monitoring the evolution of the UV index for a day, and with a frequency of one shot every 5 s. A fragment of the monitoring result can be seen in Figure 8. The figure shows how the UV index grows continuously until 15:25 when, due to the presence of clouds, the index decreases and grows sharply; other UV index drops are due to different cloudy periods during the exposure of a UV sensor.

**Figure 9:**
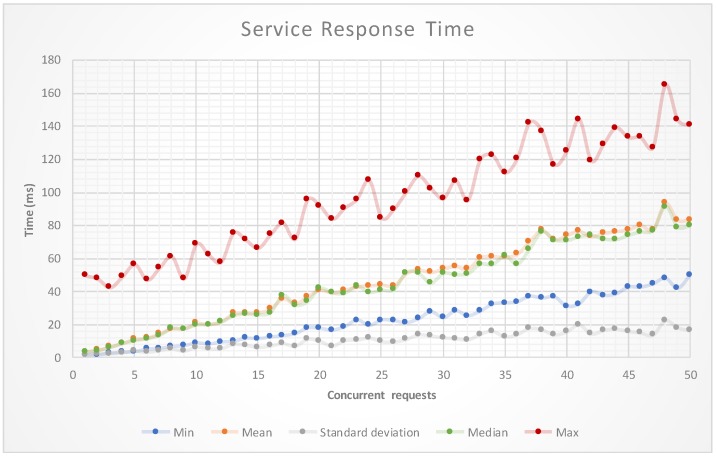
Evolution of the UV index.

In a second experiment, and with the objective of validating the sensor performance as a web service, a stress test was performed. The test consisted of a set of monitoring requests (5000 requests per sample), where the number of concurrent requests was increased from 1 to 50. Figure 9 shows how the behavior of the prototype is quite stable and with average and maximum response times adequate to the proposed objectives.

### 6.4. Artificial Intelligence Module

The monitoring flow (B in Figure 2) allows the system to periodically obtain information, both from the patient and from their environment, relevant to the treatment of their disease. The monitoring of this information is fundamental for the model, since it supposes the knowledge base. The AI module has been developed as a rule-based system using *Node.js*. We have developed multiple rules as an object-oriented decision support system, so each object is composed by a single or multiple combinations of facts. Thus, these facts are the data that stimulate the execution of the inference engine that controls the overall execution of the rules.

A knowledge-based decision support system in a clinical domain requires a high-quality knowledge base, and its construction and maintenance can consume great efforts in broad domains as chronic diseases. In this project, we have been working together with the nursing department within the Faculty of Health Sciences of the University of Alicante. They have been our experts during the development of the knowledge base, as they have a solid background in the treatment of patients with Crohn’s disease.

The inference engine maps the patients’ signs and symptoms and might suggest some lifestyle recommendations available for both physician and patient [29]. This component of a knowledge-based CDSS combines the input data and the information allocated in the knowledge base to have a result in the form of personalized lifestyle recommendations. These combinations are made with IF-THEN rules, since the knowledge is well defined by experts and does not include any probabilistic parameters in the decision-making process. The reasoning method used is forward chaining, where the knowledge is developed by a data-driven search. Thus, the method starts with the available data and uses inference rules to extract more data until it finds a rule where the antecedent (IF clause) is known to be true. When such a rule is found, the engine infers the consequent (THEN clause) resulting in new data in the form of one or more personalized recommendations. Next, to illustrate the rules definition, some examples are described.

**Example** **1.** Recommendation for a female patient in exacerbation period. This patient is willing to lose weight, since the weight reduction preference has been previously selected. The inference engine checks the body mass index and the recommended diet for patients in the exacerbation period from the knowledge base. Recommendations are made based on the preferences of this patient and her current disease situation.

**if** (disease_period = exacerbation **and**
  patient_sex = female **and**
  objective(‘weight_reduction’).selected **and**
  patient_weight/patient_height^2^ > *patient_female_optimal_bmi*)
**then** add_recommendations([
  ‘Your body mass index is higher than recommended.’,
  ‘You must reduce your weight by at least *weight_loss_value* kg.’,
  ‘You are in outbreak period: avoid foods that contain fiber and fat, legumes, vegetables and fruit.’,
  ‘You are in outbreak period: make simple meals (boiled, grilled, baked).’
])

**Example** **2.** Recommendation for a male patient in remission period. This patient is willing to reduce alcohol consumption, since the alcohol consumption reduction preference has been previously selected. The inference engine checks the physiological parameters and disease period of this patient and recommends the maximum amount of alcohol allowed in a week.

**if** (disease_period = remission **and**
  patient_sex = male **and**
  objective(‘alcohol_consumption_reduction’).selected)
**then** add_recommendations([
  ‘Eliminate alcohol consumption or reduce alcohol consumption to 210 g/weekly’
])

**Example** **3.** Recommendation for a patient in remission period. This patient is willing to increase the physical activity, since the increase activity preference has been selected previously. The inference engine checks the number of target days in a week for physical activity and the average time in a day previously introduced by the patient. Recommendations are made based on the patient preferences and his physical current status (weight and body mass index).

**if** (disease_period = remission **and**
  objective(‘increase_physical_activity’).selected **and**
  (days_with_physical_activity < *num_days_target* **or**
  average_activity_time < *average_time_target*))
**then** add_recommendations([
  ‘Your weekly preferences are in line with your physical current status’,
  ‘Your weekly activity is below your target’,
  ‘Regular practice of aerobic exercise: at least *num_days_target* days ‘,
  ‘Regular practice of aerobic exercise: moderate activity during *average_time_selected* minutes.’
])

### 6.5. Web Application

The medical web application allows the user to interact with the system. It has been developed with the AngularJS and Bootstrap framework. AngularJS supports data exchange formats in a standard way such as JavaScript Object Notation (JSON). To render the data, standard languages such as HMTL5 and JavaScript have been used, which are interpreted by the most commonly used web browsers. A set of forms and informs has been developed to allow professional to perform their configuration and monitoring tasks.

## 7. Conclusions

Our proposal is focused on the personalization of the clinical process associated with Crohn’s disease, including continuous monitoring and the analysis of patient data using artificial intelligence. To perform the personalization, a new model based on recommendations, which assists patients in their disease treatment, has been proposed. The main objectives to be achieved by the model are the inclusion of automation and patient monitoring in the clinical process, the improvement of diagnosis and treatment, and the reduction of the associated costs. The model integrates patients and physicians in a decoupled manner, and identifies three information flows: control, to supervise the process; monitoring, to acquire related information; and personalization, to support the decision-making process.

To instantiate the model, we have designed an architecture based on a services paradigm incorporating important features such as decoupling, scalability, interoperability, integration, flexibility, and reusability. The proposed architecture attempts to constitute a framework for the implementation of health services aimed at the personalization of diagnosis and treatment of Crohn’s disease. This architecture includes the acquisition of data from patients using personal or remote sensors, either manually or automatically, the storage and analysis of the data, and structured information for doctors (in the form of recommendations) to help them on the process of taking decisions for a specific patient.

To perform a functional validation, and following the architecture, we have developed a prototype integrating a set of monitoring systems to capture variables from the patient and their environment, a knowledge-based CDSS oriented to Crohn’s disease, a mobile app for patients and a web application for physicians. The prototype monitors the patient’s data, and when the physician’s criteria or objective of a patient changes, the prototype ensures the recommendations have been tailored correctly.
